# Characterization and Solid-State UV–Vis Investigations of Photoelectrocatalytically Active La_5_Cl_7_[TeO_3_]_4_, a Mixed Anion Compound with Alternating 2D Layers of Oxygen and Chlorine

**DOI:** 10.1021/acs.inorgchem.4c02392

**Published:** 2024-09-27

**Authors:** Johnny A. Sannes, Athanasios Chatzitakis, Emil H. Fro̷en, Niels Ho̷jmark Andersen, Ola Nilsen, Martin Valldor

**Affiliations:** † Centre for Materials Science and Nanotechnology (SMN), Department of Chemistry, University of Oslo, Sem Sælands vei 26, N-0371 Oslo, Norway; ‡ Centre for Materials Science and Nanotechnology (SMN), Department of Chemistry, University of Oslo, Gaustadalléen 21, NO-0349 Oslo, Norway; § Department of Chemistry, University of Oslo, Sem Sælands vei 26, N-0371 Oslo, Norway

## Abstract

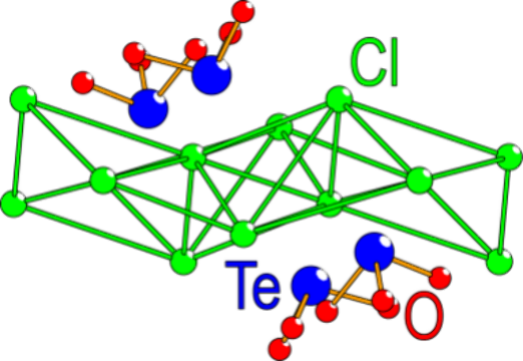

An oxide chloride, La_5_Cl_7_[TeO_3_]_4_, was synthesized using the conventional high-temperature solid-state synthesis technique in an inert atmosphere. This compound possesses a novel crystal structure that can be described with the triclinic space group *P*1̅ (No. 2) and unit cell parameters: *a* = 7.2634(3) Å, *b* = 8.1241(3) Å, *c* = 9.1993(3) Å, α = 79.373(1)°, β = 83.599(1)°, and γ = 82.511(1)°. The preference of Te(IV) to coordinate to oxygen and direct its lone pair toward the lower charged chlorine results in 2D layers of both oxygen and chlorine, alternating along the crystallographic *b*-direction. Homoleptic coordination, solely to oxygen, and heteroleptic coordination to oxygen and chlorine are observed for lanthanum, forming layers connected through edge-sharing polyhedra. In the crystal structure, two distinct tellurium positions are observed, with three close Te–O distances, emphasizing an active lone pair. The compound has been investigated by solid-state UV–vis measurements, and a band gap of 3.44 eV has been determined by DFT calculations. Detailed photoelectrochemical measurements clearly indicate that the title compound is photoelectrocatalytically active, showing an n-type behavior. Raman spectroscopy confirms that complex tellurite ions are present in the crystal structure; several observed bands can be assigned to Te–O stretching, reflecting the relatively low crystallographic symmetry of the title compound.

## Abstract 2

La_5_Cl_7_[TeO_3_]_4_, a novel crystal structure with three different rare-earth coordinations and active lone pairs, with potential for optical applications.

## Introduction

Combining two or more different monatomic anions has proven successful in finding novel materials. This is a clear distinction from the past, where the focus was mainly on varying the cation lattice by incorporating different cations. Using multiple anions leaves the experienced chemist with another tool for materials design, including rare coordinations and possible unique properties.^1,2^ Some important and interesting examples are the novel structure of Ba_3_Fe_2_O_5_Cl_2_^3^ and the promising ionic conductors Li_3_O*X* (*X* = Cl, Br),^4,5^ all obtained by introducing multiple anions.

The search for low-dimensional atomic lattices remains a contested topic within inorganic chemistry, with one recent approach being the combination of p-block elements containing a stereochemically active lone pair such as Pb(II), Bi(III), and Te(IV) and mixed anions. This approach has led to multiple novel structures such as Ni_5_(TeO_3_)_4_*X*_2_ (*X* = Cl, Br),^6^ CuSbTeO_3_Cl_2,_^7^ and SrTe_2_FeO_6_Cl.^8^ The first two compounds are examples of layered structures, and the third contains a rare anion ordering consisting of 1D zigzag chains of Cl surrounded by oxygen.^6−8^

The transition from fossil fuel to green, renewable energy sources, such as wind, hydrothermal, and solar energy, is one of the most significant challenges to date, and the search for novel materials to aid in this process is crucial for its success. A promising approach to aid in this transition is photocatalytic water splitting, which produces hydrogen and oxygen from water using sunlight as the energy source. This is done using a semiconductor with a suitable band gap and likely a catalyst to enhance the reaction rate. One promising candidate for this task is TiO_2_, published by Fujishima and Honda in 1972, which was the first material used in photocatalytic water splitting.^9^ This semiconductor has a band gap of 3.2 eV, corresponding to UV light, meaning that most of the energy released by the sun is not utilized.^10^ Hence, materials with slightly smaller band gaps and high chemical stability are needed.

One of the most apparent applications of mixed anion compounds to date is as photocatalysts,^11,12^ driven by how the band gap can be finely tuned by substituting the anions. Some of the most known examples are oxide nitrides, where the band gap is between the pure oxides and nitrides, explained by the ionic character of the *M*–O bonds compared to the *M*–N bonds. Additionally, La-containing compounds are interesting for potential doping with luminescent lanthanoids.^13^ This work reports on La_5_Cl_7_[TeO_3_]_4_, an oxide chloride with a novel crystal structure and a band gap of 3.44 eV.

## Materials and Methods

### Synthesis

La_5_Cl_7_[TeO_3_]_4_ was first observed as single crystals after a synthesis combining Fe_2_O_3_ (VWR Chemicals, 99%), LaCl_3_ (LaCl_3_*xH_2_O, Sigma-Aldrich 99.9%, vacuum-dried in-house; the synthesis was later successfully reproduced using ultradry LaCl_3_, provided by Thermo Scientific, ultradry, 99.99%), and TeO_2_ (Acros Organics 99+%) in a 1:1:1 molar ratio was performed. The reactants were ground using an agate mortar and pestle inside an argon-filled glovebox (GS Glovebox Systemtechnik GmbH, O_2_ and H_2_O < 2 ppm). Once thoroughly mixed, the mixture was transferred to a corundum crucible placed inside a silica ampule. To avoid excessive overpressure inside the ampule during heating, the pressure was reduced to approximately 0.2 bar. This was done using a septum and syringe inside the glovebox before sealing the ampule using a hydrogen–oxygen torch. The silica ampule, containing the corundum crucible filled with the sample, was heated inside a muffle furnace at 600 °C using a heating rate of 5 °C min^−1^ for 10 h before cooling to room temperature at an ambient rate. The sample used for further characterization was synthesized by mixing TeO_2_, LaCl_3_, and La_2_O_3_ (Molycorp, 99.99%) in stoichiometric amounts corresponding to La_5_Cl_7_[TeO_3_]_4_ similarly as described above; however, a temperature of 700 °C was used. In both synthesis attempts, tiny metallic particles, believed to be metallic Te, could be observed inside the ampules.

### Powder and Single-Crystal X-ray Diffraction (pXRD and SC-XRD)

pXRD data were measured using a Bruker D8 DISCOVER with Bragg–Brentano geometry and equipped with a Ge(111) Johansson monochromator, selecting only Cu*K*α_1_ (λ = 1.5406 Å) radiation and a LYNXEYE detector. A flat plate XRD sample holder, fitted with a Si crystal and coated with silicone grease, was used as the sample holder. Single-crystal data were acquired from a Bruker D8 VENTURE single-crystal diffractometer with a Mo*K*α Incoatec microfocus X-ray source and a PHOTON 100 detector. The Rietveld refinement of the pXRD data and the structure solution of the SC-XRD data were performed using JANA2020.^14^ To investigate the purity of the synthesized powder sample and provide an additional indication that the structure model obtained by SC-XRD is correct, a Rietveld refinement of the pXRD data was performed. The background and peak profiles were fitted using a 20-term Chebyshev polynomial and the pseudo-Voigt function, applying GU, GW, LX, and LY, respectively. To account for peak asymmetry at low angles, Howard (Boole’s rule) was included in the refinement. As unrestricted isotropic atomic displacement parameters lead to negative values for some of the atoms, all ADP values were restricted to be identical during the Rietveld refinement. To achieve accurate estimations of the standard deviations of the structural parameters, Berar–Lelann corrections were applied.^15^

### Scanning Electron Microscopy and Energy-Dispersive X-ray Spectroscopy (SEM and EDX)

SEM images were captured using a Hitachi SU8230 field-emission scanning electron microscope (FESEM) with an electron current and acceleration voltage of 10 μA and 1 kV, respectively. The elemental composition was determined using an XFlash 6|10 energy-dispersive X-ray spectroscopy (EDX) detector from ten measurements performed on different crystallites, using an electron current and acceleration voltage of 30 μA and 25 kV, respectively.

### Ultraviolet–Visible Spectroscopy (UV–Vis Spectroscopy)

Absorbance data were acquired from reflectance measurements using the Kubelka–Munk method,^16^ which were measured using a Flame-S spectrometer from Ocean Optics, equipped with a halogen and deuterium lamp and optical fibers and with the incident light normal to the sample surface and the detector at a 45° angle.

### Density Functional Theory (DFT)

DFT calculations were performed utilizing the Vienna Ab initio Software Package (VASP).^17,18^ All calculations used the Perdew–Burke–Ernzerhof (PBE)^19^ generalized gradient approximation (GGA) exchange-correlation functional with projected augmented-wave (PAW)^20^ pseudopotentials.

The plane-wave cutoff energy was set to 550 eV, with an ionic relaxation convergence criterion of 0.01 eV Å^–1^ and a self-consistent-field energy cutoff of 10^–6^ eV. Both the ionic positions and the unit cell were allowed to relax. Relaxation of the crystal structure utilized a gamma-centered 5 × 4 × 5 grid to sample the Brillouin zone. Integration over the Brillouin zone utilized the tetrahedron method with Blöchl corrections for all calculations except the band structure; the latter utilized Gaussian smearing with a smearing width of 0.02 eV. The high-symmetry path for the band structure was determined with the SeeK-path tool.^21,22^

The alignment of the band edges of the title compound relative to the vacuum energy was determined by electrostatic alignment of a surface calculation with the previously obtained bulk results.^23^ The surface calculation cell consisted of a six-layer slab, with the crystal terminated perpendicular to the *b*-axis across the Cl-connections while keeping the La–O–Te bonds intact, as this appeared to be the least complicated surface to consider. The surface slab used the relaxed parameters from the bulk calculations, with the atomic positions being kept static throughout the calculations. The slabs were separated by a 20 Å vacuum layer.

### Photoelectrochemical Measurements

The working electrode containing the mixed anion compound La_5_Cl_7_[TeO_3_]_4_ was prepared by adding 50 mg of powder in a mixture of 1.25 mL of water, 0.65 mL of isopropanol, and 15 μL of 5 wt % Nafion solution, followed by sonication for 30 min to obtain a homogeneous ink. Then, 50 μL of ink was drop-casted on FTO glass (Ossila, TEC 10 FTO coated glass, unpatterned, 20 × 15 × 1.0 mm^3^) covering an area of approximately 1 cm^2^. The electrode was left to dry in ambient air and used as received in a typical three-electrode cell. A saturated calomel electrode (SCE) and Pt foil were used as reference and counter electrodes, respectively. All photoelectrochemical (PEC) tests were conducted in 0.5 M Na_2_SO_4_ by an Ivium Vertex potentiostat/galvanostat under 1 sun simulated solar light (Newport Oriel LCS-100 solar simulator equipped with a 100 W ozone-free xenon lamp and an AM 1.5G filter). The light intensity was calibrated by a monocrystalline Si PV reference cell (Newport 91150 V-KG5). All potentials were corrected against the reversible hydrogen electrode (RHE) taking into account that water electrolysis takes place thermodynamically at 1.23 V vs RHE. Therefore, the potentials were corrected vs RHE according to the Nernst Equation shown in eq 1.
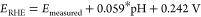
1where *E*_RHE_ is the potential against the RHE and *E*_measured_ is the measured potential against the SCE.

### Raman Spectroscopy

The spectrum acquired was measured using a T64000 Horiba Jobin-Yvon spectrograph in a single mode via a confocal microscope. A power of 1 mW laser (Nd:YVO_4_, λ = 532.1 nm) at the sample, focused with an Olympus LMPlanFl 50*x*/0.5 objective, required exposures of 10 s averaged six times to get a spectrum of good quality. The detector was a nitrogen-cooled Symphony II open electrode 256 × 1024 CCD with a pixel size of 2.54 × 2.54 μm. The 900 rules/mm grating served as a dispersion element and, combined with an entrance-slit width of 100 μm and a focal length of 640 mm, resulted in an observed spectral width of 5.1 cm^–1^. The scale of the spectrum was corrected against a spectrum of paracetamol, leading to an uncertainty of ±0.5 cm^–1^.

## Results

The structural parameters obtained by the SC-XRD measurement are summarized in Table 1.

**Table 1:** Obtained Structure Parameters for La_5_Cl_7_[TeO_3_]_4,_ As Determined by SC-XRD

chemical formula	La_5_Cl_7_[TeO_3_]_4_
fw (g mol^–1^)	1645.1
temperature	ambient
λ (Å)	0.71073
crystal system	triclinic
space group	*P*1̅ (No. 2)
*a* (Å)	7.2634(3)
*b* (Å)	8.1241(3)
*c* (Å)	9.1993(3)
α (°)	79.373(1)
β (°)	83.599(1)
γ (°)	82.511(1)
*V* (Å^3^)	526.84(3)
*Z*	1
density (g cm^–3^)	5.1851
*R*(obs)/*R*(all) (%)	2.96/6.77
*R*_*w*_(obs)/*R*_*w*_(all) (%)	4.50/5.25
GOF(obs)/GOF(all)	1.12/1.09
diff Fourier peak/hole (e Å^–3^)	3.51/–3.47
index ranges	–12 ≤ *h* ≤ 13, −15 ≤ *k* ≤ 14, −15 ≤ *l* ≤ 15
no. of obs. refl.	51,960
CSD	2339402

The atomic coordinates for La_5_Cl_7_[TeO_3_]_4_, determined by SC-XRD, are shown in Table 2.

**Table 2:** Atomic Coordinates for La_5_Cl_7_[TeO_3_]_4_

atom, Wyckoff, *x*, *y*, *z*, *U*_11_, *U*_22_, *U*_33_, *U*_12_, *U*_13_, *U*_23_	La1	2*i*	0.93885(2)	0.20627(2)	0.61108(2)
			0.00555(7)	0.00705(7)	0.00625(7)
			–0.00101(5)	–0.00063(5)	–0.00127(5)
	La2	1*f*	0.5	0	0.5
			0.0073(1)	0.0156(1	0.0058(1)
			–0.00165(8)	–0.00109(8)	–0.00235(8)
	La3	2*i*	0.71104(2)	0.11594(2)	0.06432(2)
			0.00967(7)	0.01304(8)	0.00596(7)
			–0.00472(6)	0.00165(6)	–0.00355(6)
	Te1	2*i*	0.19544(3)	0.19605(2)	0.22053(2)
			0.00762(8)	0.00644(8)	0.00602(8)
			–0.00150(6)	–0.00161(6)	–0.00005(6)
	Te2	2*i*	0.38325(3)	0.29333(2)	0.75256(2)
			0.00749(8)	0.00627(8)	0.00871(8)
			–0.00056(6)	–0.00240(6)	–0.00108(6)
	Cl1	2*i*	0.7822(1)	0.2657(1)	0.31756(8)
			0.0142(3)	0.0148(3)	0.0125(3)
			0.0003(3)	–0.0057(3)	–0.0043(3)
	Cl2	2*i*	0.2255(1)	0.43238(9)	0.44520(9)
			0.0202(4)	0.0092(3)	0.0146(3)
			–0.0035(3)	–0.0023(3)	–0.0010(3)
	Cl3	2*i*	0.9618(1)	0.3450(1)	0.88490(9)
			0.0226(4)	0.0136(3)	0.0135(3)
			–0.0053(3)	0.0020(3)	–0.0044(3)
	Cl4	1*e*	0.5	0.5	0
			0.0468(9)	0.0250(7)	0.0433(9)
			–0.0175(6)	–0.0203(7)	0.0049(6)
	O1	2*i*	0.1190(3)	0.0133(3)	0.1479(2)
			0.015(1)	0.012(1)	0.0079(9)
			–0.0043(8)	0.0007(8)	–0.0041(8)
	O2	2*i*	0.2543(3)	0.1228(3)	0.7027(2)
			0.0069(9)	0.0095(9)	0.0094(9)
			–0.0018(7)	–0.0023(7)	–0.0023(8)
	O3	2*i*	0.1520(3)	0.0761(2)	0.4192(2)
			0.0083(9)	0.0087(9)	0.0056(9)
			–0.0024(7)	0.0006(7)	–0.0002(7)
	O4	2*i*	0.4476(3)	0.1341(3)	0.2402(2)
			0.0069(9)	0.013(1)	0.0067(9)
			–0.0001(7)	–0.0001(7)	–0.0016(8)
	O5	2*i*	0.4278(3)	0.1536(3)	0.9341(2)
			0.020(1)	0.010(1)	0.0074(9)
			–0.0028(8)	–0.0061(8)	0.0007(8)
	O6	2*i*	0.5997(3)	0.2186(3)	0.6431(2)
			0.0053(9)	0.013(1)	0.019(1)
			0.0001(8)	0.0021(8)	–0.0022(9)

**Figure 1:**
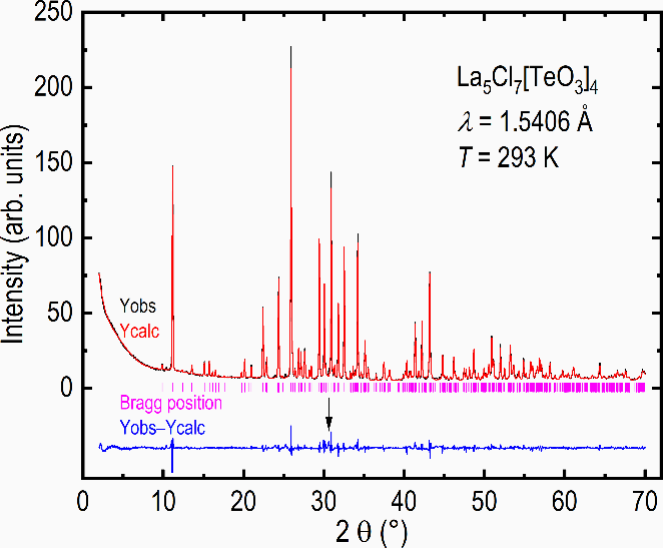
Rietveld refinement of the pXRD data measured for La_5_Cl_7_[TeO_3_]_4_. The observed data are shown in black (Yobs), the calculated diffractogram is shown in red (Ycalc), the difference curve is shown in blue (Yobs–Ycalc), and the expected positions of the Bragg peaks are shown as purple vertical lines (Bragg position). The most significant extrinsic reflection, corresponding to an impurity, is positioned where a LaOCl reflection is expected and is marked using an arrow.

To further indicate that the structure model acquired by SC-XRD was correct and to check the purity of the synthesized sample, a Rietveld refinement was performed. The resulting fit is shown in Figure 1. The largest extrinsic reflection, corresponding to an unknown impurity, is shown by using an arrow. Upon further inspection of many of the extrinsic reflections, a good match is observed with LaOCl, a likely impurity given its stability.^24^

The resulting structure parameters, obtained from the Rietveld refinement of the pXRD data, are summarized in Table S1, in the Supporting Information (SI); however, the atomic positions, determined by SC-XRD and pXRD, are very similar, with minor discrepancies for the oxygen positions.

**Figure 2:**
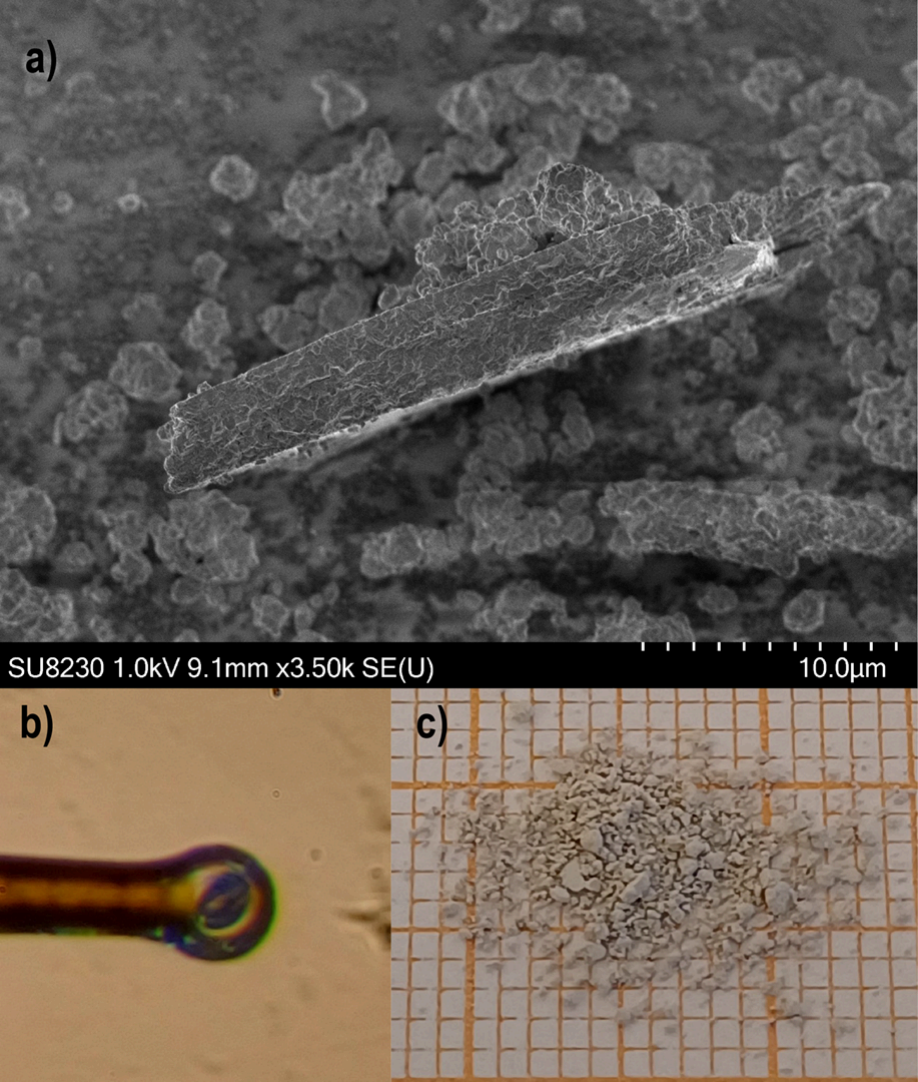
(a) SEM image of some small crystallites of La_5_Cl_7_[TeO_3_]_4_; the entire scale bar corresponds to 10 μm. (b) Picture of the La_5_Cl_7_[TeO_3_]_4_ crystal, held in place by a sample holder with a diameter of 20 μm, used in the SC-XRD measurement, from which the crystal structure could be solved. (c) Picture of the polycrystalline sample of La_5_Cl_7_[TeO_3_]_4_.

The crystal morphology was investigated using SEM, and an image highlighting some small crystallites of La_5_Cl_7_[TeO_3_]_4_ is shown in Figure 2a. From the SEM image, irregular morphologies can be observed, which are not unexpected for triclinic crystals. The La_5_Cl_7_[TeO_3_]_4_ crystal used in the SC-XRD measurement is shown in Figure 2b, held in place by an SC-XRD sample holder with a diameter of 20 μm. The synthesized polycrystalline sample is a light-gray powder, as shown in Figure 2c. The EDX measurements of ten different crystallites result in an averaged elemental composition of La_5.0(2)_Te_3.8(1)_O_12_Cl_6.1_,^2^ assuming 12 oxygen atoms and that the correct oxidation state is Te(IV). This is a reasonable assumption when considering the Te coordination and structure, as discussed in greater detail in later sections.

**Figure 3:**
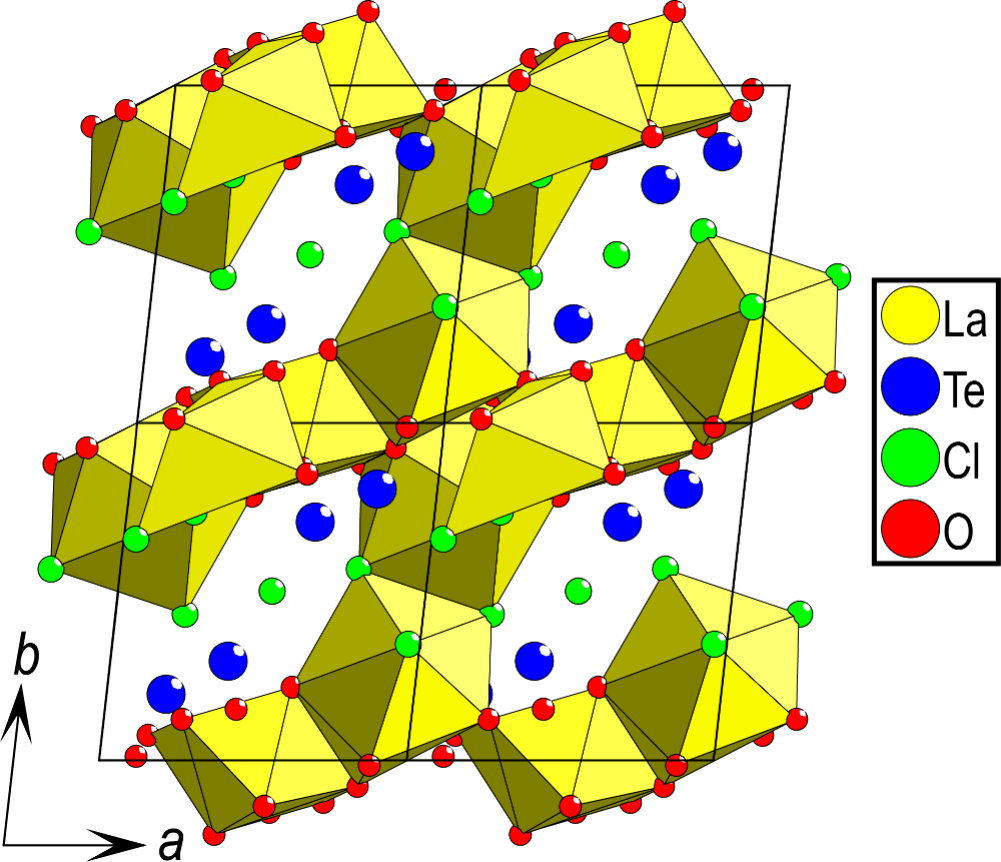
Four unit cells of La_5_Cl_7_[TeO_3_]_4_ highlight the almost 2D nature of the sample; however, edge-sharing between the La polyhedra is observed between the layers.

Considering the unit cell of La_5_Cl_7_[TeO_3_]_4_, a 3D structure can be observed with 2D layers of lanthanum connected through edge-sharing between the La polyhedra, as observed in Figure 3, where four unit cells in the *a–b* plane are shown.

**Figure 4:**
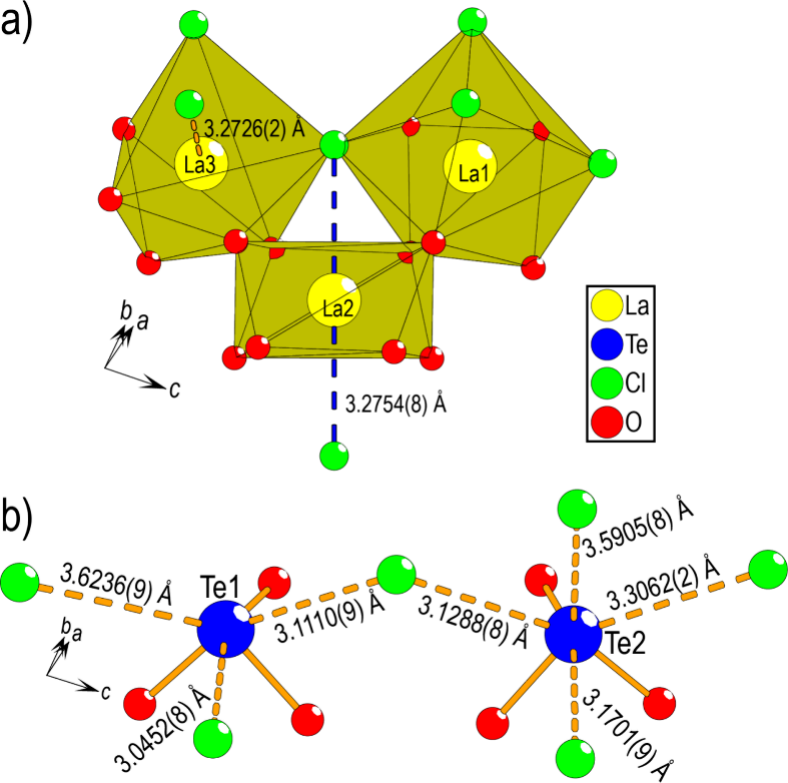
(a) Three distinct La atoms observed for La_5_Cl_7_[TeO_3_]_4_. In the case of La2, homoleptic coordination to eight oxygen atoms is observed, while in the case of La1 and La3, heteroleptic coordination to five oxygen atoms and two and four chlorides, respectively, is observed. Additional chlorine atoms close to the limit of what can be considered bonding are shown by using dashed lines. (b) There are two Te coordinations, with three close oxygen atoms, forming typical [TeO_3_*E*] units. The dashed lines represent the closest Te–Cl distances.

Three distinct La atoms are observed in the structure, as shown in Figure 4a. La2 has homoleptic coordination to eight oxygen atoms, which varies between 2.485(2) and 2.677(2) Å, while La1 and La3 possess heteroleptic coordination to five oxygen atoms and two or four chlorides, respectively, for which the La–O distances vary between 2.440(2)–2.609(2) and 2.372(2)–2.622(2) Å and the La–Cl distances vary between 2.9718(9)–3.0483(8) and 2.9300(8)–2.9416(9) Å for La1 and La3 respectively. Comparing it to La–O and La–Cl interatomic distances found in La_2_O_3_ and LaCl_3_, one finds very similar distances of 2.396(1)–2.773(1) and 2.972(1)–2.995(1) Å, respectively.^25,26^ Both edge- and corner-sharings are observed between the different La polyhedra. Additionally, in the case of La2 and La3, two or one additional chlorine can be observed at 3.2754(8) and 3.2726(2) Å, respectively. This La–Cl distance is considerably longer than that observed in LaCl_3_. However, using Brown’s operational definition of a bond, contributing more than 4% of the cation valence,^27^ it is strictly bonding, although it is close to the limit of 3.33 Å for La–Cl. These La–Cl interatomic distances are shown as dashed lines in Figure 4a.

In the case of Te, two distinct positions are observed, as shown in Figure 4b. In both cases, three short Te–O distances between 1.855(2) and 1.919(2) Å are observed, forming typical [TeO_3_*E*] units, where *E* denotes the lone pair of Te(IV). These Te–O interatomic distances are similar to related structures such as SbTeO_3_Cl^28^ and Ni_5_(TeO_3_)_4_Cl_2_^6^_._ The next Te–O distance is 3.02(1) Å and is therefore not considered within the primary coordination. The closest observed Te–Cl interatomic distance is 3.05(1) Å for Te1 and is from bond valence sum (BVS) considerations exactly on the limit of what can be considered bonding. The different Te–Cl interatomic distances are shown as dashed lines in Figure 4b. BVS values have been calculated for the distinct atomic sites and can be found in Table S2 in SI. For all the atomic sites, reasonable values are observed, except for the three chlorine atoms, and the deviation is especially large for Cl4, where a BVS of 0.28 is determined (the closest La–Cl and Te–Cl interatomic distances observed for Cl4 are 3.2726(2) and 3.3062(2) Å, respectively). This observation is not uncommon for halides in oxide halides, as pointed out previously in the literature, and suggests that the anion takes the role of a counterion in the structure.^29^ The calculations were performed using *R*_0_(Te–O) = 1.977, *R*_0_(Te–Cl) = 2.37, *R*_0_(La–O) = 2.172, *R*_0_(La–Cl) = 2.545, and *B* = 0.37.^30,31^

**Figure 5:**
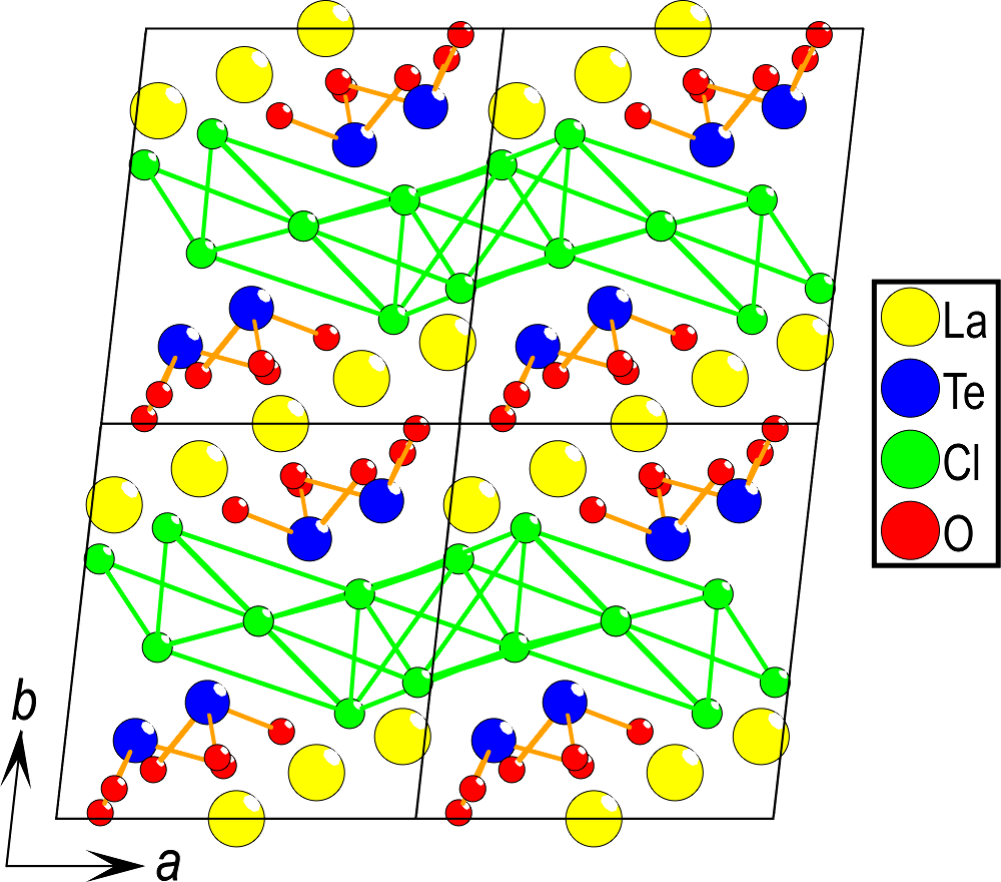
Four unit cells of La_5_Cl_7_[TeO_3_]_4_ in the *a*–*b* plane highlight how the chloride forms 2D layers separated from the oxygen layers. It is also apparent that the lone pair on Te(IV) is directed toward the chloride layers.

Considering only the anion ordering, 2D chloride layers can be observed, clearly separated from the oxygen layers, as shown in Figure 5. Interestingly, the [TeO_3_*E*] units are directed with the lone pair of Te(IV) toward the chloride layers.

**Figure 6:**
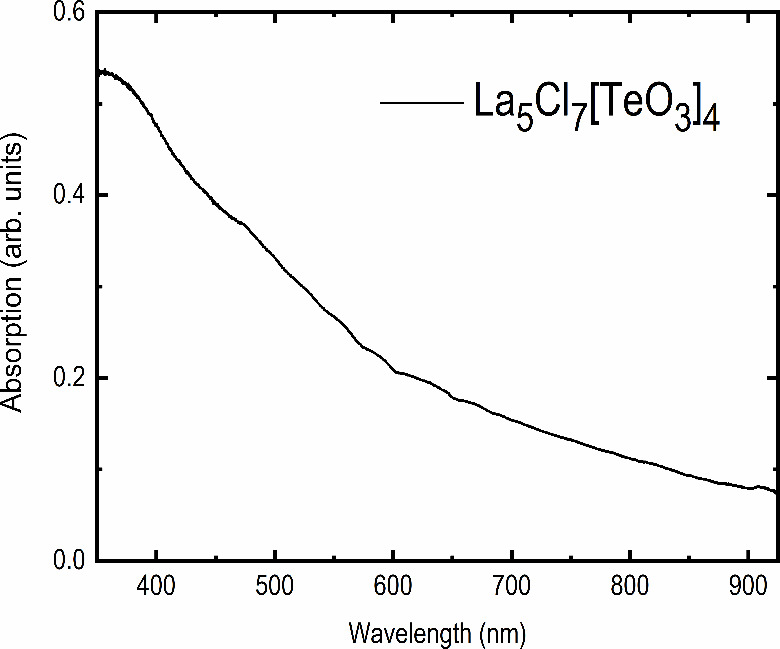
Absorption spectrum for La_5_Cl_7_[TeO_3_]_4_, acquired using the Kubelka–Munk approach on reflectance data.

From the light-gray color of the sample, a band gap corresponding to photons with energy close to visible light seemed apparent. Absorption data, acquired by applying the Kubelka–Munk method to reflectance data, were obtained and are shown in Figure 6. In order to estimate the band gap of the material, a Tauc plot was constructed by plotting (α*hv*)^1/*y*^ versus *hv*.^32^ However, for both *y* = 1/2 and *y* = 2, corresponding to a direct and indirect transition, respectively, an unreasonably low value for the band gap was achieved, as shown for *y* = 1/2 in Figure S1. The estimated band gap from the Tauc plot of *E*_g_ ≈ 2.5 eV is considerably lower than expected from the color of the sample. The exact reason for this mismatch is still unknown; however, it may originate from defects in the material and/or the presence of some Te metal, a likely impurity given the EDX results (see Figure S2 in SI).

**Figure 7:**
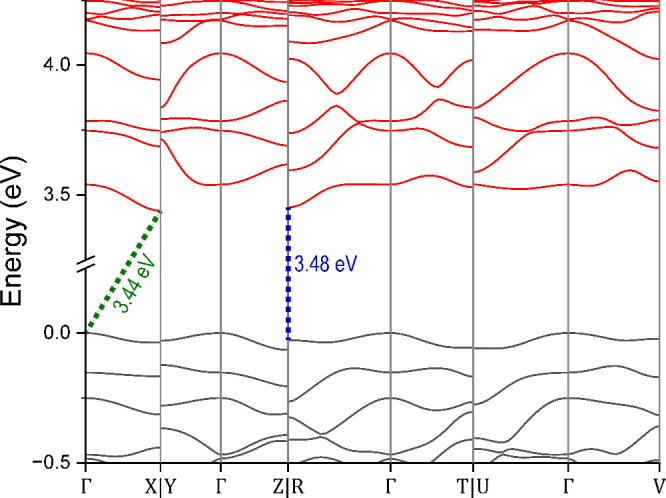
Band structure of La_5_Cl_7_[TeO_3_]_4_. The valence bands are shown in black, while the conduction bands are shown in red. The blue and green stapled lines show the minimum band gap corresponding to direct and indirect transitions, respectively.

To gain further insight into the electronic transition and to calculate the band gap of the material, DFT calculations were performed, and the band structure of La_5_Cl_7_[TeO_3_]_4_ is shown in Figure 7. The calculations show that the compound is a semiconductor with several band gap transitions in the range of about 3.4–3.5 eV. The minimum band gap corresponding to direct and indirect transitions is shown by blue and green stapled lines in Figure 7, respectively.

**Figure 8:**
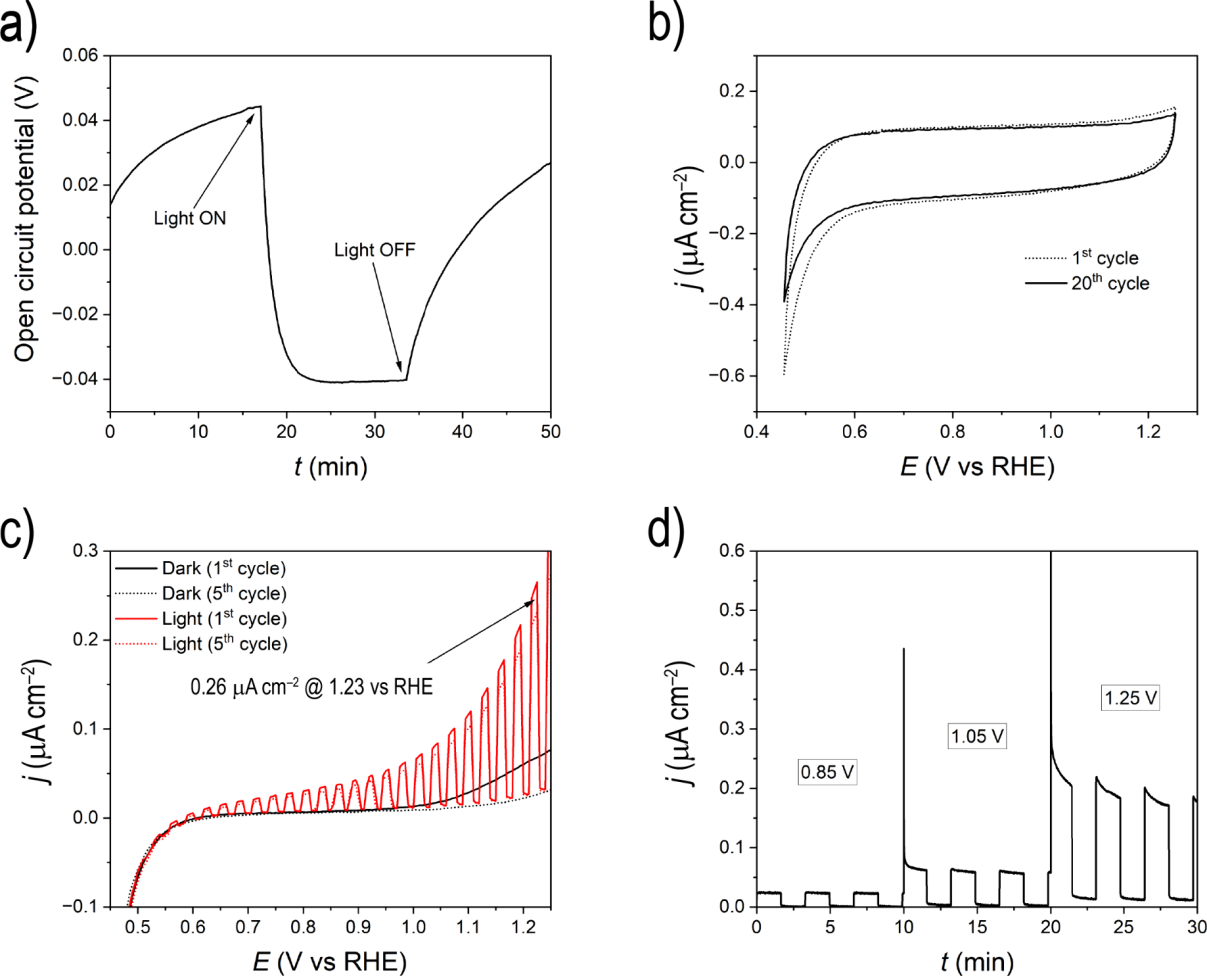
PEC measurements in 0.5 M Na_2_SO_4_ (pH = 6.9). (a) OCP measurement under light and dark conditions. (b) CV measurements at a scan rate of 50 mV s^–1^. The first and the twentieth stabilized cycles are given. (c) LSV measurements in the dark and under chopped illumination conditions at a scan rate of 5 mV s^–1^. The first and the fifth stabilized cycles are given. (d) Chronoamperometric experiment at three applied potentials, 0.85, 1.05, and 1.25 V, under dark and chopped illumination conditions. The potentials mentioned in the figure are vs RHE.

The band gap energy of about 3.44 eV is indicative of the semiconducting nature of the mixed anion compound La_5_Cl_7_[TeO_3_]_4_. To investigate a possible photoelectrocatalytic activity, a series of PEC measurements were conducted, which are presented in Figure 8. The open circuit potential (OCP) measurement (Figure 8a) shows that upon illumination with solar simulated light (100 mW cm^–2^), there is a negative shift in the potential, which is a typical behavior for an n-type semiconductor. This is because of a negative shift in the Fermi level (E_F_) under illumination, indicating the presence of a built-in voltage due to band bending in the space charge region of the material.^33,34^ With this in mind, La_5_Cl_7_[TeO_3_]_4_ was regarded as a possible photoanode electrode in a PEC cell. Cyclic voltammograms (CV) in the potential window between ∼0.4 V vs RHE and 1.3 V vs RHE (Figure 8b) show no distinct redox peaks but only capacitive currents, which can be assigned to the double-layer capacitance in the electrode/electrolyte interface. This behavior shows the inertness of the material under the studied conditions. As expected by the OCP measurement of Figure 8a, the material is photoactive, and the linear sweep voltammograms (LSV) shown in Figure 8c are typical for an n-type photoelectrocatalyst.^35^ The generation of anodic photocurrent upon illumination that increases with increasing potential is observed, explained by the increase in the band bending with increasing potential, leading to increased charge carriers’ separation. This is a clear indication of occurring oxidation reactions at the electrode/electrolyte interface upon illumination due to the generation of electron holes. Finally, steady-state measurements at three applied potentials (0.85, 1.05, and 1.25 V vs RHE) under dark and illumination conditions were carried out, and the results are shown in Figure 8d. The photocurrent densities at the three chosen potentials correspond very well with the expected photocurrent densities from the LSV curves of Figure 8c, indicating steady-state conditions during the LSV measurements too. Furthermore, both the LSV and chronoamperometric measurements indicate a photocatalytically stable mixed anion compound in the studied time frame. In conclusion, all the PEC measurements provided direct evidence of the photoelectrocatalytic activity of La_5_Cl_7_[TeO_3_]_4_ as an n-type photoelectrocatalyst (photoanode) in an aqueous, pH-neutral electrolyte.

**Figure 9:**
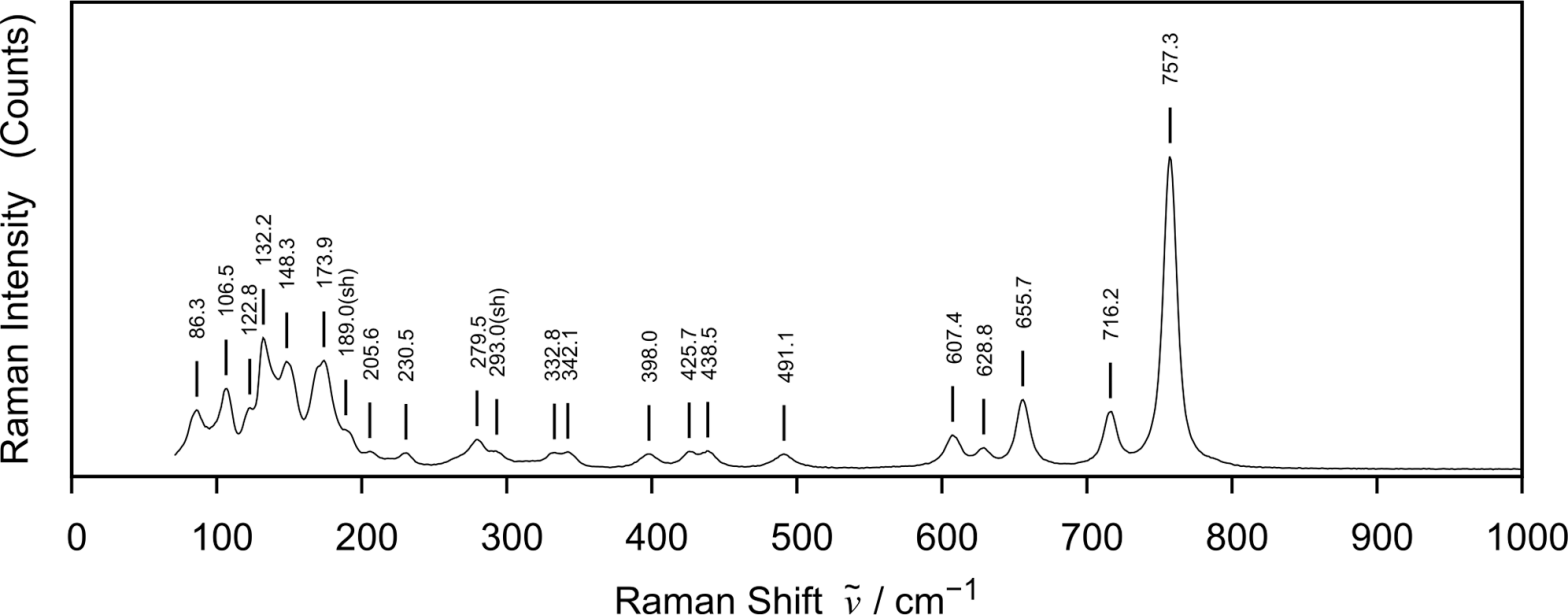
Raman spectrum measured for La_5_Cl_7_[TeO_3_]_4_.

The novel compound La_5_Cl_7_[TeO_3_]_4_ has been investigated by Raman measurements, and the results are shown in Figure 9.

The free tellurite ion in water solutions, TeO_3_^2–^, has a trigonal pyramidal structure and is characterized by four vibrational modes, which are all Raman active. The nondegenerate symmetric modes ν_1_(*A*_1_) = 758 cm^–1^ and ν_2_(*A*_1_) = 364 cm^–1^ represent stretching and bending, respectively, where the first typically is the most intense band. The modes ν_3_(*E*) = 703 cm^–1^ and ν_4_(*E*) = 326 cm^–1^ are both double degenerate and assigned to asymmetric stretching and bending, respectively.^36^ According to the site-group approach, these will split when the *C*_3v_ symmetry of tellurite is broken, while the symmetric modes will not. However, according to the crystal structure found in this work, two distinct types of tellurite are identified, and these are also bound to chloride ions at longer distances. Taking into account the chloride ions, one could say the fac-trichloro surrounded tellurite Te2-atom rather has a distorted capped antiprism geometry with the Te(IV) lone pair pointing along the *C*_3_ axis toward the chlorides. Despite this, *C*_3v_ symmetry remains a fair local structure approximation. The tellurite Te1-atom coordinated by three oxygen atoms and only two chloride ions leaves it without any nearby suitable symmetry.

Among the highest frequencies noted for Te(IV)–Cl bonds is the 374 cm^–1^ band of Te_2_Cl_9_.^37^ In the same paper, solid H[TeCl_4_OH] has a band at 667 cm^–1^ assigned to the Te–O bond stretching, while the Te–Cl-related bands all fall below 289 cm^–1^. Asawa et al.^38^ studied LaCl_3_ as a crystalline solid and demonstrated that first-order Raman bands could be observed in the range from 108 to 219 cm^–1^. Based on these studies, it would be highly unlikely to expect the involvement of chloride ions in vibrational modes above 374 cm^–1^.

Gopinath and Brown^39^ did measurements of several lanthanum-based oxides, and they observed first-order Raman bands for La_2_O_3_ in the range from 99 to 413 cm^–1^. Haeuseler,^40^ who studied LaOCl crystals, observed Raman bands from 125 to 525 cm^–1^ and assigned the highest frequency to oxygen movement.

Despite the paper by Frost et al.,^41^ we assign here the peak at 757.3 cm^–1^ to the symmetric stretching mode of both the Te1 and Te2 tellurite entities because these do not differ grossly in structure. More distant chloride ions do not influence the frequency enough for the band to split, but it could well be composed of two contributions. Nevertheless, the asymmetric stretching modes are more sensitive to local environmental differences. Consequently, we propose all the bands ranging from 716.2 to 607.4 cm^–1^ to be representative of asymmetric stretching.

The group of bands ranging from 491.1 to 398.0 cm^–1^ are lost, likely due to tellurite bending and La–O stretching, while those from 342.1 cm^–1^ and downward include Cl–La and Cl–Te stretching modes and composite bending of various kinds. The lowest frequencies are most likely dominated by lattice modes, requiring detailed simulations to be assigned accurately. A table of peak positions, relative intensities, and peak widths can be found in Table S3 in SI.

## Discussion

The obtained structure model from SC-XRD has been validated by the observation that an almost X-ray pure sample can be synthesized when starting from the composition suggested by the structure model. A similar composition is further corroborated by EDX. However, some challenges were encountered during the synthesis of this compound. First, small metallic particles can be observed inside the ampule when performing the synthesis, which, based on EDX performed on small crystallites observed in SEM, seems to be metallic tellurium (see Figure S2 in SI). The second challenge arises when one attempts to synthesize the compound using various LaCl_3_ batches as a starting reactant. When using LaCl_3_ (Sigma-Aldrich, 99.9% anhydrous beads), additional peaks from different phases could be observed in addition to La_5_Cl_7_[TeO_3_]_4_ from a pXRD measurement. However, when using a batch that was dried in-house, an almost X-ray pure sample was obtained. The two starting reactants were analyzed by pXRD, and no significant difference was observed. Further investigations revealed that an X-ray pure sample could be synthesized when using ultradry LaCl_3_ (Thermo Scientific, ultradry, 99.99%), indicating that the synthesis could potentially be sensitive to small amounts of water during the synthesis. A comparison of the three La_5_Cl_7_[TeO_3_]_4_ samples synthesized using the dried in-house, anhydrous, or ultradry LaCl_3_ is shown in Figure S3 in SI.

When performing the DFT calculations, the structural relaxation resulted in lattice parameters that showed excellent agreement with the experimental values. The obtained lattice parameters consistently overestimated the length of the unit cell sides, but the difference was less than 2% for all axes. The unit cell angles all agreed with the experimental values within 1%. The result shows that the compound is nominally an indirect semiconductor with a band gap of 3.44 eV. The narrowest transition occurs between the Γ-X symmetry points. A second transition with a nearly equal band gap of 3.45 eV occurs between the Γ-R symmetry points. The narrowest direct band gaps are only slightly wider, with a width of about 3.48 eV. These direct transitions occur at both the X and the R symmetry points.

**Figure 10:**
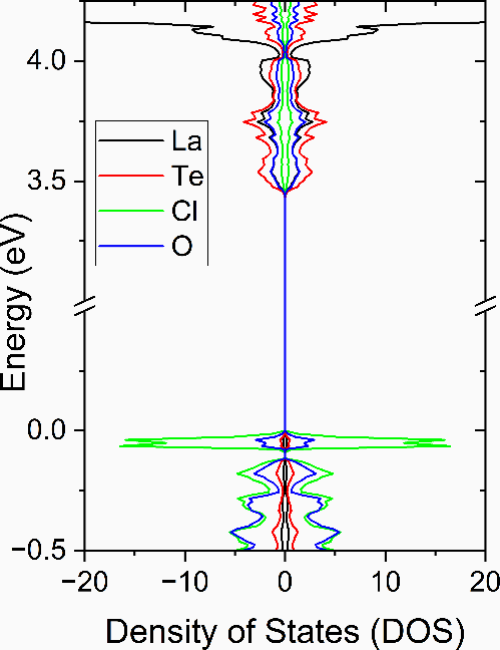
Atom projected density of states for La_5_Cl_7_[TeO_3_]_4_.

For all the transitions under consideration, direct and indirect, the valence band maximum (VBM) consists predominantly of Cl-3*p* states with a secondary contribution from O-2*p* states, while the conduction band minimum (CBM) exhibits mixed character; Te-5*p* states dominate the band edge, but there is a secondary contribution of mixed La-4*f* and -5*d* states and mixed O-3*s* and -3*p* states, making the compound a charge-transfer semiconductor. The atom projected density of states for La_5_Cl_7_[TeO_3_]_4_ is shown in Figure 10. Additionally, from the DFT calculations, the band edges for La_5_Cl_7_[TeO_3_]_4_ were determined to be at −7.0 and −3.6 eV for VBM and CBM, respectively. The values straddle the redox potentials necessary for splitting water.^42^ Compared with the first catalyst used for water splitting, TiO_2_, the band edges correspond to a type-II alignment.^43^

**Figure 11:**
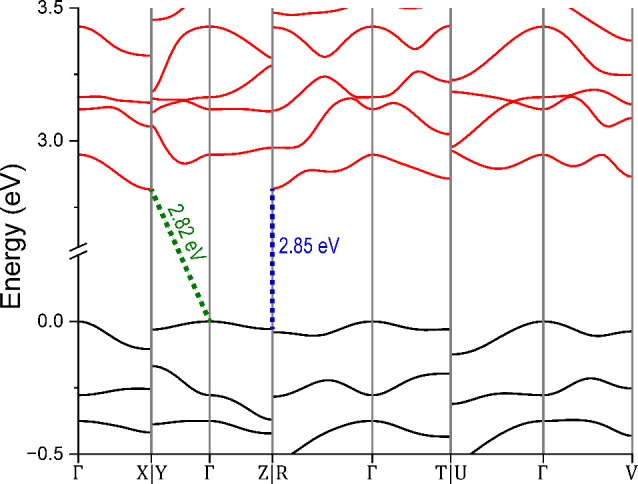
Band structure of the theoretical La_5_Br_7_[TeO_3_]_4_. The valence bands are shown in black, and the conduction bands are shown in red. The blue and green stapled lines show the minimum band gap corresponding to direct and indirect transitions, respectively.

DFT calculations reveal that the band gap is 3.44 eV, corresponding to light with a wavelength slightly below the visible part of the electromagnetic spectrum; however, given the multianion nature of the compound, tuning of the band gap could be possible by substituting the anions. For instance, the band gap is expected to increase if Cl is substituted by F and decrease if Cl is substituted by Br. The difference in band gap is explained by the fact that F and Br have higher and lower electronegativity than Cl, increasing and decreasing the band gap, respectively. For many oxide chlorides, the substitution of Cl with Br is possible, see, for instance, Sr_2_FeO_3_*X* (*X* = Cl, Br),^44^ Sr_3_Fe_2_O_5_*X*_2_ (*X* = Cl, Br),^44^ and Ba_2_Co_4_*X*O_7_ (*X* = Cl, Br),^45^ which is promising for the observation of the, so far, theoretical La_5_Br_7_[TeO_3_]_4_. To investigate how the band gap would be influenced by substituting Cl^–^ with Br^–^, a DFT calculation of the yet theoretical La_5_Br_7_[TeO_3_]_4_ was performed, and the calculated band structure of La_5_Br_7_[TeO_3_]_4_ is shown in Figure 11. As expected from the lower electronegativity of Br, as compared to Cl, the band gap is significantly lowered from 3.44 and 3.48 eV to 2.82 and 2.85 eV for the indirect and direct transition, respectively. A band gap of 2.85 eV corresponds to light with a wavelength of λ ≈ 435 nm, within the visible region, which is beneficial for potential application as a photocatalyst as more energy is available from natural sunlight. For comparison, using the AM1.5G solar spectrum, a photocatalyst with a band gap of 3.44 eV (λ ≈ 360 nm) will only be able to utilize the UV part of the solar spectrum corresponding to ∼2% of the solar energy; however, a photocatalyst with a band gap of 2.85 eV (λ ≈ 435 nm) could utilize ∼9%. Attempts were made to calculate the properties of the F-homologue. The relaxed atomic structure appeared physically implausible due to the small size of the F^–^ ion being incompatible with the halide positions. If La_5_F_7_[TeO_3_]_4_ exists, it likely crystallizes in a different crystal structure.

The combination of p-block elements containing stereochemically active lone pairs and mixed anions has led to the discovery of multiple novel compounds, as mentioned in the introduction; however, it also allows for some control of the anion lattice, as shown for this compound. The p-block element coordinates to the higher valent oxide anions and directs the lone pair toward the lower valent halide ion. In the case of La_5_Cl_7_[TeO_3_]_4_, this is shown by a clear layering of the anions, with the tellurium atoms located in between the layers. This was also the case in SrTe_2_FeO_6_Cl;^8^ however, in this case, this led to a 1D zigzag chain of chlorine. Similar behavior is observed in Cu_2_Te_2_O_5_*X*_2_ (*X* = Cl, Br),^46^ where the anion lattice can be explained by tetrahedra of Cl or Br atoms surrounded by [TeO_4_*E*] units. Using p-block elements containing stereochemically active lone pairs and mixed anions with differences in oxidation states seems like a promising synthesis route for interesting low-dimensional anion lattices.

## Conclusions

An oxide chloride, La_5_Cl_7_[TeO_3_]_4_, with a novel crystal structure, has been synthesized by a conventional solid-state synthesis technique, utilizing an Ar-filled glovebox and a silica ampule to control the reaction atmosphere. The crystal structure can be described in the triclinic symmetry *P*1̅ (No. 2) and unit cell parameters: *a* = 7.2634(3) Å, *b* = 8.1241(3) Å, *c* = 9.1993(3) Å, α = 79.373(1)°, β = 83.599(1)°, and γ = 82.511(1)°. The compound has been investigated using solid-state UV–vis spectroscopy, and DFT calculations disclosed several transitions, both direct and indirect, with minimum gap widths of 3.48 and 3.44 eV, respectively. Potential photoelectrocatalytic activity was also investigated by several PEC measurements under 1 sun solar simulated light, clearly indicating that La_5_Cl_7_[TeO_3_]_4_ is an n-type photoelectrocatalyst. As DFT calculations indicate a significant narrowing of the band gap for the Br-homologue, as compared to the Cl-homologue, from 3.44 and 3.48 eV to 2.82 and 2.85 eV for the indirect and direct transition, respectively, further work into chemical flexibility, especially regarding anion substitution, could prove interesting as a potential candidate for photocatalytic water splitting. Alternatively, the novel crystal structure offers three distinct La positions within the lattice, meaning that doping with luminescent lanthanoids such as Tb(III) and Eu(III) is a likely possibility from a chemical perspective and could be interesting for light-emitting or optical conversion applications.

## Supporting Information Available

Obtained structure parameters for La_5_Cl_7_[TeO_3_]_4_ as determined by Rietveld refinement of the pXRD data; calculated BVS values; Tauc plot; EDX measurements; elemental composition; peak positions, relative intensities, and peak widths from the Raman measurement (PDF)
