# Supplementary material for: Characterization and Solid-State UV–Vis Investigations of Photoelectrocatalytically Active La5Cl7[TeO3]4, a Mixed Anion Compound with Alternating 2D Layers of Oxygen and Chlorine
Source: Inorg Chem. 2024 Sep 27;63(40):18632–41. doi: 10.1021/acs.inorgchem.4c02392 (PMC11462499; doi:10.1021/acs.inorgchem.4c02392)
Supplement: Supplementary file 1 — ic4c02392_si_001.pdf [file ic4c02392_si_001.pdf]

Supporting Information

Characterization and Solid-State UV-VIS investigations of Photoelectrocatalytically Active $La_5Cl_7[TeO_3]_4$, a mixed anion compound with alternating 2D layers of oxygen and chlorine

Johnny A. Sannes[1], Athanasios Chatzitakis[2], Emil H. Frøen[1], Niels Højmark Andersen[3], Ola Nilsen[1] and Martin Valldor[1]*

[1]Centre for Materials Science and Nanotechnology (SMN), Department of Chemistry, University of Oslo, Sem Sælands vei 26, N-0371 Oslo, Norway

[2]Centre for Materials Science and Nanotechnology (SMN), Department of Chemistry, University of Oslo, Gaustadalléen 21, NO-0349 Oslo, Norway

[3]Department of Chemistry, University of Oslo, Sem Sælands vei 26, N-0371 Oslo, Norway

Corresponding Author

*Johnny Sannes, email: j.a.sannes@kjemi.uio.no

The resulting structure parameters, determined by the Rietveld refinement of the pXRD data, are summarized in Table S1 for $La_5Cl_7[TeO_3]_4$. The atomic positions, determined by SC-XRD and pXRD, are very similar, with minor discrepancies for the oxygen positions.

Table S1. The obtained structure parameters for $La_5Cl_7[TeO_3]_4$ as determined by Rietveld refinement of the pXRD data.

chemical formula	$La_5Cl_7[TeO_3]_4$
fw (g mol^{-1})	1645.1
temperature	ambient
λ (Å)	1.5406
crystal system	triclinic
space group	$P\bar{1}$ (No. 2)
a (Å)	7.2700(2)
b (Å)	8.1354(3)
c (Å)	9.2106(3)
α (°)	79.385(2)
β (°)	83.600(2)
γ (°)	82.576(2)
V (Å^3)	528.79(3)
Z	1
density (g cm^{-3})	5.166
R(obs)/*R*(all) (%)	2.82/2.86
R_w(obs)/R_w(all) (%)	3.62/3.65
GOF(all)	0.2039

diff Fourier peak/hole (e Å^{-3})	0.41/–0.52		
atom, Wyckoff, *x*, *y*, *z*, U_{iso}	La1	2*i*	0.9405(9)
	0.2063(7)	0.6123(8)	0.008(1)
	La2	1*f*	0.5
	0	0.5	0.008(1)
	La3	2*i*	0.7106(8)
	0.1158(7)	0.0647(8)	0.008(1)
	Te1	2*i*	0.195(1)
	0.1945(8)	0.2203(9)	0.008(1)
	Te2	2*i*	0.3854(9)
	0.2960(8)	0.7534(9)	0.008(1)
	Cl1	2*i*	0.775(3)
	0.277(2)	0.316(3)	0.008(1)
	Cl2	2*i*	0.224(3)
	0.433(3)	0.440(3)	0.008(1)
	Cl3	2*i*	0.965(3)
	0.347(3)	0.888(3)	0.008(1)
	Cl4	1*e*	0.5
	0.5	0	0.008(1)
	O1	2*i*	0.123(7)
	0.012(6)	0.152(6)	0.008(1)
	O2	2*i*	0.277(7)
	0.107(6)	0.713(6)	0.008(1)
	O3	2*i*	0.142(7)
	0.083(6)	0.429(6)	0.008(1)
	O4	2*i*	0.444(7)
	0.159(5)	0.218(6)	0.008(1)
	O5	2*i*	0.423(7)
	0.172(6)	0.912(5)	0.008(1)
	O6	2*i*	0.598(7)
	0.223(5)	0.633(5)	0.008(1)

To investigate the bonding strength between the different atoms in $La_5Cl_7[TeO_3]_4$, Bond Valence Sum (BVS) calculations were performed for all the distinct atom sites, and the results are summarized in Table S2.

Table S2. Calculated BVS values for the distinct atom sites in $La_5Cl_7[TeO_3]_4$ using

R_0(Te – O) = 1.977, R_0(Te – Cl) = 2.37, R_0(La – O) = 2.172, R_0(La – Cl) = 2.545 and B = 0.37

Atom 1	Atom 2	Interatomic Distance (Å)	BVS	Atom 1	Atom 2	Interatomic Distance (Å)	BVS
Te1	O4	1.8548	1.39	O1	Te1	1.9034	1.22
	O1	1.9034	1.22		La3	2.5137	0.40
	O3	1.9195	1.17		La1	2.6083	0.31
	Cl1	3.0452	0.16			**Sum**	**1.92**
		**Sum**	**3.94**	O2	Te2	1.9171	1.18
Te2	O6	1.8649	1.35		La1	2.4969	0.42
	O5	1.8729	1.32		La3	2.6227	0.30
	O2	1.9171	1.18		La2	2.6716	0.26
		**Sum**	**3.85**			**Sum**	**2.15**
La1	O6	2.4392	0.49	O3	Te1	1.9195	1.17
	O2	2.4969	0.42		La1	2.5067	0.40
	O3	2.5067	0.40		La1	2.539	0.37
	O3	2.539	0.37		La2	2.6769	0.26
	O1	2.6083	0.31			**Sum**	**2.20**
	Cl3	2.9719	0.32	O4	Te1	1.8548	1.39
	Cl1	2.9799	0.31		La3	2.3717	0.58
	Cl2	2.9961	0.30		La2	2.4856	0.43
	Cl2	3.0484	0.26			**Sum**	**2.40**
		**Sum**	**3.16**	O5	Te2	1.8729	1.32
La2	O4	2.4856	0.43		La3	2.4518	0.47
	O4	2.4856	0.43		La3	2.5245	0.39
	O6	2.6118	0.30			**Sum**	**2.18**
	O6	2.6118	0.30	O6	Te2	1.8649	1.35
	O2	2.6716	0.26		La1	2.4392	0.49
	O2	2.6716	0.26		La2	2.6118	0.30
	O3	2.6769	0.26			**Sum**	**2.14**
	O3	2.6769	0.26	Cl1	Te1	3.0452	0.16
	Cl1	3.2754	0.14		La3	2.9416	0.34
	Cl1	3.2754	0.14		La1	2.9799	0.31
		**Sum**	**2.77**		La2	3.2754	0.14
La3	O4	2.3717	0.58			**Sum**	**0.95**
	O5	2.4518	0.47	Cl2	La1	2.9961	0.30
	O1	2.5137	0.40		La1	3.0484	0.26
	O5	2.5245	0.39			**Sum**	**0.55**
	O2	2.6227	0.30	Cl3	La3	2.93	0.35
	Cl3	2.93	0.35		La1	2.9719	0.32
	Cl1	2.9416	0.34			**Sum**	**0.67**
	Cl4	3.2726	0.14	Cl4	La3	3.2726	0.14
		**Sum**	**2.97**		La3	3.2726	0.14
						**Sum**	**0.28**

From the absorption data acquired by utilizing the Kubelka-Munk approach on reflection data, a Tauc plot could be constructed for $La_5Cl_7[TeO_3]_4$. However, for both *y* = ½ and *y* = 2, an unreasonably low band gap was determined for $La_5Cl_7[TeO_3]_4$, as shown for *y* = ½ in Figure S1.

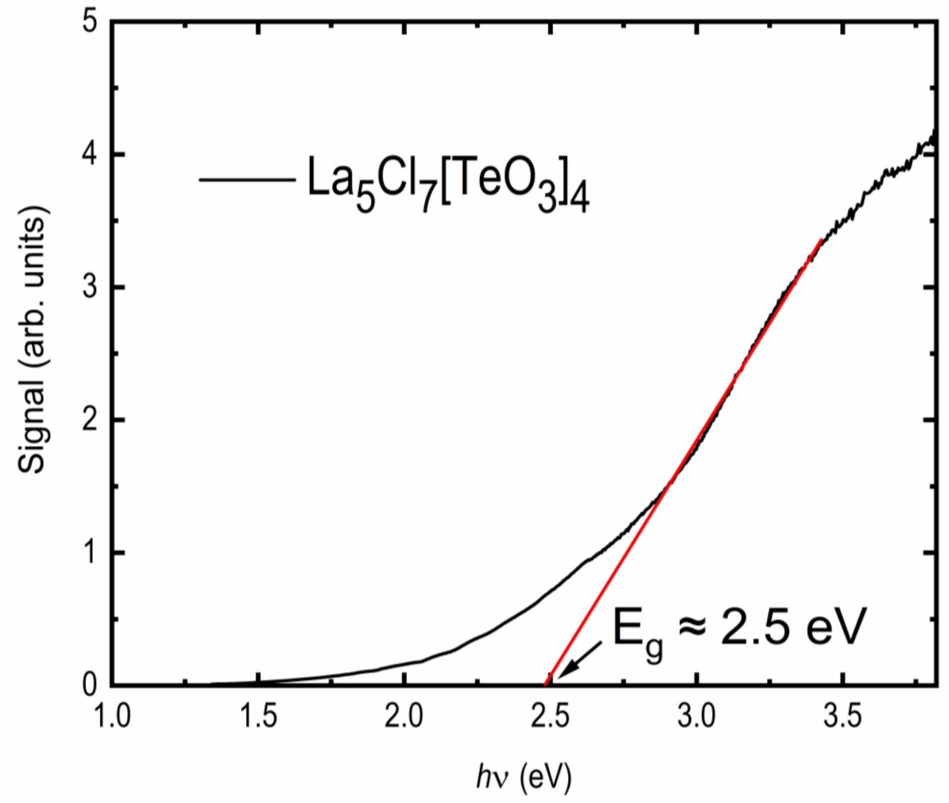

Figure S1. The constructed Tauc plot for $La_5Cl_7[TeO_3]_4$, where $(\alpha h\nu)^2$ versus $h\nu$ was plotted, resulted in a band gap of $E_g \approx 2.5$ eV. The red line has been drawn by hand.

When performing EDX measurements on the sample, small crystallites of metallic tellurium could be observed, as shown in Figure S2.

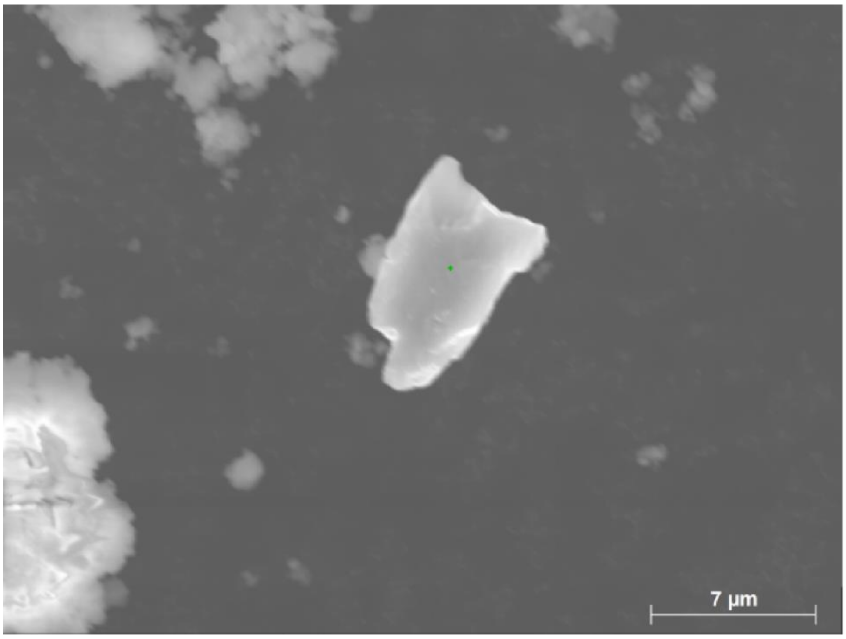

Element:	Atomic %
Carbon	33
Oxygen	5
Aluminium	19
Chlorine	1
Tellurium	43
Lanthanum	0

Figure S2. A small particle observed during the EDX measurements of $La_5Cl_7[TeO_3]_4$. The resulting elemental composition is shown in the table on the right. The carbon and aluminum are assumed to originate from the carbon tape used to hold the sample in place and the interior of the instrument, respectively. The green dot in the middle of the particle is the spot used for the EDX measurement.

The peak positions, relative intensities, and peak widths from the Raman measurement, performed for $La_5Cl_7[TeO_3]_4$, are summarized in Table S3. The bandwidths were based on the second order polynomial fit terms imitating a Gaussian function.

Table S3. The peak positions, relative intensities, and peak widths from the Raman measurement performed for $La_5Cl_7[TeO_3]_4$. The bandwidths were based on the second order polynomial fit terms imitating a Gaussian function.

Peak position (cm^{-1})	Relative intensity	Peak width (cm^{-1})
757.3	100	6.2
716.2	20.3	8.1
655.7	24	6.4
628.8	8.9	8.7
607.4	12.9	6.6
491.1	7	9.5
438.5	8	7.4
425.7	7.8	11.4
398	7	12.8
342.1	7.7	8.2
332.8	7.4	12.8
293.0(sh)	8.9	8
279.5	11.6	7.3
230.5	7.4	10.1
205.6	7.8	8.3
189.0(sh)	14.9	8
173.9	36.2	8.7
148.3	35.8	9.7
132.2	43.3	6.2
122.8	21.5	7.5
106.5	27.5	8.3
86.3	20.8	6.8

A comparison between the diffractogram measured for the two $La_5Cl_7[TeO_3]_4$ samples, synthesized using the dried in-house or ultra-dry $LaCl_3$, is shown in Figure S3a. From the comparison, it can be observed that the two samples are essentially identical, with only some small differences indicated using asterisks in the figure. However, the anhydrous sample is significantly different, as shown in Figure S3b. It should be noted that the synthesis utilizing the anhydrous $LaCl_3$ shown in the figure has been synthesized using a temperature of 600 °C, as opposed to 700 °C. However, other synthesis attempts using the dried in-house $LaCl_3$ at similar temperatures did not yield similar results, indicating that it is the different reactant and not the temperature that is the reason for the difference.

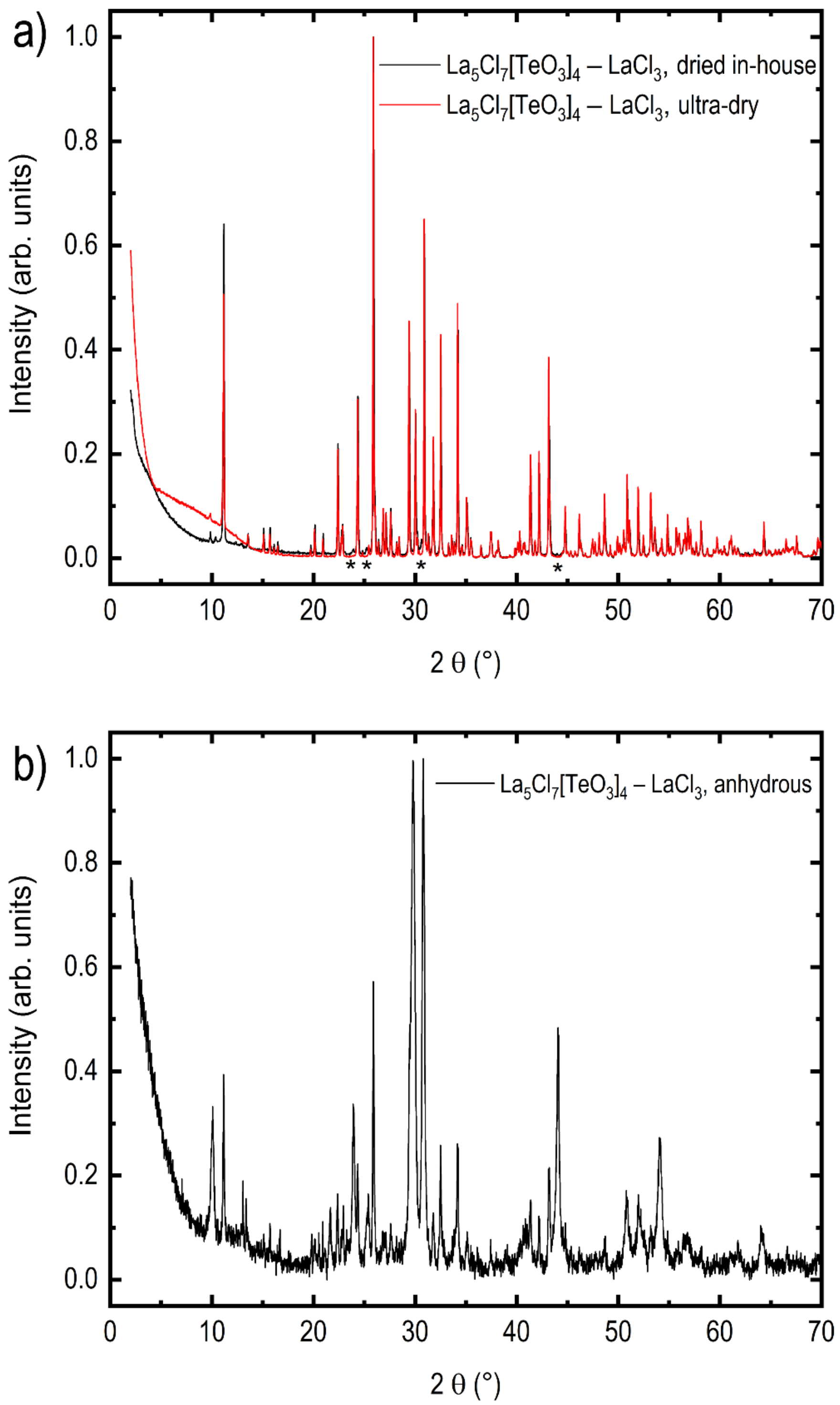

Figure S3. a) A comparison of pXRD data for two $La_5Cl_7[TeO_3]_4$ samples synthesized using the dried in-house (black) $LaCl_3$ and ultra-dry (red) $LaCl_3$. Only very small differences between the two diffractograms can be observed, as shown using asterisks in the figure. b) pXRD of a $La_5Cl_7[TeO_3]_4$ sample synthesized using anhydrous $LaCl_3$, clearly resulting in additional phases.
